# Let the team fix it?—Performance and mood of depressed workers and coworkers in different work contexts

**DOI:** 10.1371/journal.pone.0256553

**Published:** 2021-10-14

**Authors:** Martin Vollmann, Christiane Schwieren, Margarete Mattern, Knut Schnell

**Affiliations:** 1 Alfred Weber Institute of Economics, Heidelberg University, Heidelberg, Germany; 2 Department of General Psychiatry, Heidelberg University, Heidelberg, Germany; 3 Asklepios Medical Centre Göttingen, University Medicine Göttingen, Göttingen, Germany; University of Glasgow, UNITED KINGDOM

## Abstract

Depression in the workplace is a significant factor for reduced personal well-being and productivity. Consequently, this has negative effects on the economic success of the companies in which depressed people are employed. In addition, the economy has to deal with the significant burden of this illness on the health system. In this paper, we investigated how different working contexts—working in a group or individually—influenced depressed individuals towards higher or lower well-being and productivity. We examined this using a laboratory experiment. In this setting, we were also able to analyze how, in turn, a depressive individual impacted the productivity and affective situation of their workgroup, reflecting the company perspective. The experimental design mimicked the very basic processes of a workplace in a stylized way. We used two distinct samples: subclinically and clinically depressed, both working in a group with healthy controls. As expected, we found generally lower performance in the clinically depressed sample, but in the subclinically depressed sample, we only found this in the individual work context. In contrast to our expectations, the performance of subclinically depressed individuals working in groups with healthy controls was even higher than that of healthy controls in homogenously healthy groups. The performance of the entire group with a depressed member was lower for the sample with clinically manifested depression, while the performance of groups with a subclinically depressed participant was significantly higher than the performance of homogeneously non-depressed control groups. We discuss our results with a focus on the design of workplaces to both re-integrate clinically depressed employees and prevent subclinically depressed employees from developing major depression.

## Introduction

Depression in the workplace is a major factor for the reduction of personal well-being and productivity. Unipolar depressive disorders are widespread [1] and as Mathers and Loncar [2] pointed out, they will most likely become the second leading cause of Disability-Adjusted Life Years (DALYs) by 2030 behind HIV/AIDS. One reason for this is the failure to support and re-integrate individuals with depression into the workforce, which has a negative effect on the course of the disorder [3, 4]. From the management perspective within a company, depression means a significant reduction of performance and presence in the workplace [5–10], which can cause a loss of knowledge holders who are pivotal for the success of entire projects [11]. In the larger scope, depression derived disability is also a problem for national economies by means of expenses for treatment costs and sickness benefits. Sobocki et al. [12] estimated that depression resulted in a total annual cost of 118 billion Euro in 2004 for Europe.

To avoid the perceivable consequences of depressive disorders, companies should be able to take measures to i.) prevent the incidence of depressive disorders in their employees and ii.) avoid relapse in workers after treatment. To do so, it is essential to know how the work environment can be designed to reduce stress, positively influence the mood and productivity of employees with a risk of depression and, similarly, prevent those with prevalent milder forms of depression from dropping out of the workforce. To address these questions, we apply an experiment to test how the *interpersonal context* of the workplace—working in a stylized group setting or individually—impacts the mood and productivity of clinically and subclinically depressed and non-depressed workers and the groups they work in.

Current research suggests that the typical performance reductions found in people suffering from depression are caused by an interaction of reduced cognitive resources [e.g., 13, 14] and altered motivational schemas with indifference or a shift from a behavioral approach towards avoidance [15–17]. The associated cognitive schema has been described by Beck’s cognitive model of depression [18]. In this model, depressive cognition is characterized by a negative view of oneself, the people around and the future. A contemporary update of this model by Beck [19] included three elements of dysfunctional cognition: biased attention, biased processing and biased memory. All these biases are supposed to create a feedback loop which supports the initiation or sustainment of an episode of depression [20]. Following this model and the current findings, excessive work-related demands, perceived reduction in performance and resulting negative feedback should increase the aversive perception of the work context, reinforce depressive assumptions and result in the increased severity of the depressive syndrome. Thus, depressed individuals should be more open to react to the negative feedback of group members in comparison to positive feedback, which initiates a vicious cycle. A similar argument, from an economic perspective, has been made by de Quidt & Haushofer [21], who model depression as a negative shock that leads to pessimistic beliefs about the returns to one´s own effort, potentially initiating a vicious cycle that depends on the reaction to that shock.

It has been demonstrated that performance in depressed and non-depressed workers generally depends on job characteristics like occupational status and psychosocial working conditions [22, 23]. Plaisier et al. [24] found that high job support, high job control, fewer work hours, being self-employed and having a high-skilled job positively affected psychopathology, absenteeism and work performance. Another factor that can influence individual well-being and productivity is the *interpersonal context* of the workplace. According to the mentioned models of depression, interaction with other employees can either be a source of resilience or a stress factor causing further mood dysregulation and subsequent loss of productivity. Therefore, the core question of this research concerns whether or not working in groups pushes depressed employees further into a negative mood state and how the productivity of the whole group is affected by the depressed team member. From the perspective of employers, understanding the effects of such interactions could help prevent the initial emergence of major depression and relapse during reintegration attempts and the resulting enduring loss of the workforce. While previous studies, reaching back to the work of Isen and coauthors in the 1980s [25, 26] and the work of Oswald and colleagues [27] mostly focused on positive mood (induction) and its effects on decision making and productivity, we look at the other, negative side of the coin, focusing on clinical and sub-clinical depression.

We are the first to systematically study in a controlled experimental context how the mood and productivity of a depressed individual are influenced by the working context in a stylized group setting and how, in turn, a depressed individual affects the productivity and mood of the group. Moreover, we compare this scenario to the same task done individually to be able to give recommendations to managers on how to optimize the interpersonal work context for depressed employees. We use a highly stylized work setting often used in experimental economics to study various aspects of workplace contexts that allows us to elicit effort using either team- or individual incentives, and to study the effect of highly standardized within-group feedback provision. In addition, we focus on two situations to be managed from a company perspective: prevention of dropout from the workforce caused by developing depression and avoiding the relapse of reintegrated workers. Thus, we look at two distinct samples i.) individuals on the brink of depression (“Subclinically Depressed”) and ii.) with depression under treatment (“Clinically Depressed”), both working in a stylized work setting with healthy controls. As in real life working groups, the group members were not informed if one of their colleagues was suffering from depression. Unlike in laboratory studies of the effects of stress on behavior, using, for example, the Trier Social Stress Test (for an overview see Kudielka et al. [28]) we cannot induce depression in the lab, but select participants who have been diagnosed with depression. The focus is thus on the effect of a stylized working context on people with a pre-existing condition and how the working context affects mood productivity in this group of people.

The structure of the remaining paper is as follows. Section 2 presents the experimental design. Section 3 contains the hypotheses and section 4 presents the results divided by the two samples. The last section discusses the results and contains concluding remarks.

## Experimental design

### Setup

To understand how the interpersonal context—groupwork (“Group”) and individual work (“Single”)—influences the performance and mood of individuals on the brink of depression (“Subclinically Depressed”) and with depression under treatment (“Clinically Depressed”), we used two distinct samples. On the one hand, we used a standard student sample (“Subclincial Sample”), which underwent an on-site self-classification based on the Beck Depression Inventory-II [29]. Depending on the result, participants were categorized as “Subclinically Depressed” or “Healthy Control.” On the other hand, we invited a sample consisting of clinically diagnosed major depressive disorder (MDD) patients that were close to being reintegrated into the workforce and a matched healthy control group (“Clinical Sample”).

In the “Group” setting, each group consisted either of four “Healthy Controls” or of three “Healthy Controls” and one “Subclinically/Clinically Depressed” participant. In the “Single” setting, participants worked individually on the same task. Table 1 summarizes all four experimental treatments. This design fulfills the purpose of isolating the direct effect of depression on performance from the effect of performing in a group setting, including social evaluation by peers. The categorization of depressed or healthy (i.e., not depressed) was not disclosed to the participants to avoid the potential effects on behavior.

**Table 1:** Experimental treatments.

	Group	Single
**Subclinically/Clinically Depressed**	3 Healthy Controls 1 Depressed Participant	1 Depressed Participant
**Healthy**	4 Healthy Controls	1 Healthy Participant

### Participant recruitment and depression assessment

Participants in the “Subclinical Sample” were recruited from the subject pool of the experimental laboratory of Heidelberg University (AWI-Lab). The experiment was organized and the sample recruited with the software hroot [30]. The participants underwent an on-site self-classification with the Beck Depression Inventory-II (BDI-II) [29]. Based on this, participants were categorized as “Subclinically Depressed” (N = 89) or “Healthy Control” (N = 250). The cutoff was chosen based on the standard norms where scores >13 indicate at least a mild depressive syndrome. Even if the BDI-II was not designed for diagnostic purposes, its classification accuracy has been shown in multiple studies [31–33], and it is regularly used to categorize student samples [e.g., 34].

Participants in the “Clinical Sample” were recruited in two different ways. “Clinically Depressed” participants were recruited from the Clinic of General Psychiatry of the Heidelberg University Hospital or the Asklepios Medical Center Göttingen and participated in agreement with their attending physician. Thereby, major depressive disorder (MDD) diagnosed by expert raters according to ICD 10 criteria (F32.1, F32.2, F33.1 or F33.2) had to be the main diagnosis. Exclusion criteria comprised a set of other mental disorders, comprising organic mental disorders, addictions (except nicotine), schizophrenic disorders, bipolar disorders, post-traumatic stress disorders, personality disorders, attention deficit syndromes, autism disorders and impairment of intelligence. Other psychiatric comorbidities did not constitute exclusion criteria. Furthermore, participants had to fulfill the following inclusion criteria. They had to be between 20–60 years old and worked at least part-time no longer than twelve months before the inpatient or day-clinic treatment. Furthermore, they had to be close to being released from a stationary treatment or a day-clinic treatment. This resulted in N = 24 clinically depressed participants.

The “Healthy Control” (N = 108) participants were recruited by announcements on the internet and at different locations within the city of Heidelberg. To match these participants with the treatment group, they had to meet the same inclusion criteria (age, professional status, gender) except that they never suffered from a psychiatric illness. To ensure that the potential participants met these conditions, a student assistant conducted a short telephone screening. The screening questions were based on the short form of the Camberwell Assessment of Need (CAN) [35], from which those were chosen that could detect all mental disorders defined as exclusion criteria.

During the recruitment and the conduction of the experiment, all requirements to ensure medical confidentiality were met and documented. Written ethics approval has been granted by the Ethics committee of the Faculty of Behavioral and Empirical Culture Studies of Heidelberg University on April 12, 2018 under the approval number Schwi-2018/1-2.

### Procedure group setting

In the “Group” setting, the procedures differed slightly between the two samples (“Subclinical” and “Clinical”). For the “Subclinical Sample,” the participants arrived at the laboratory and were randomly placed according to the standard procedure of the AWI-Lab. The group matching was made by an algorithm based on the BDI-II Scores of the participants that were assessed computer-based at the beginning of each session. For the “Clinical Sample,” all participants assembled in front of the Clinic of General Psychiatry of the Heidelberg University Hospital (respectively Asklepios Clinic Göttingen). Afterwards, they were collectively guided to the experimental laboratory, where they received an identification (ID) number, which ensured that the questionnaires from the pre-screening could be connected to the entries in the group task on the computer. For the experiments in Göttingen, we used a mobile laboratory consisting of four laptops and cubicles, which matched in size and appearance with the AWI-Lab to ensure comparability. Clinically depressed participants received a special ID number, which made it possible to identify them later in the data set. The distribution of the numbers and the numbers themselves were designed in a way that complete anonymity was ensured. After arrival at the laboratory, participants could choose a computer, and the experimenter started the experiment. Since the participants in the “Clinical Sample” were not registered in the participant pool of the AWI-Lab, written informed consent was obtained before the experiment started.

The experiment itself was divided into four parts. In the first part, the participants either answered the BDI (“Subclinical Sample”) or entered their ID number (“Clinical Sample”). Thereafter, the instructions were displayed on the screen. The instructions were the same for all participants and provided them with complete information for the upcoming task.

In the second part, the participants got to know the task by playing two practice periods that allowed participants to become acquainted with the setup [cf. 36]. The results of this had no impact on their payoff, and they were not analyzed.

Afterward, 12 payment periods followed, which were identical to the practice periods, only differing in the time limit. The time to solve the task in the practice periods was shorter to avoid a strong learning effect.

Finally, all participants had to fill out a demographic questionnaire. When the participants had finished the computer tasks and filled out the questionnaire, they received their payment and left the laboratory. In addition, participants in the “Clinical Sample” filled out several psychological and psychiatric questionnaires for clinical assessment, including the Holt and Laury risk elicitation task [37], on a pen and paper basis. A complete list of the questionnaires used and the Holt and Laury task can be found in the Online Supplementary Information. Three “Clinically Depressed” and four “Healthy Controls” did not fill out their questionnaires.

The task itself was structured in the following way. Participants had to solve simple arithmetic problems, i.e., adding or subtracting numbers by one or two digits. These mathematical tasks are commonly used in experimental research to evoke real effort without strong demands on intellectual abilities [38–40]. Each period was started by a five second countdown followed by a 60 second time limit to answer as many arithmetic problems as possible. After the end of the 60 seconds, the screen immediately changed, and no further entries were possible. On the next screen, the participants were informed of several aspects of performance. In the “Group” setting, they learned i.) the total number of correct answers by the group, ii.) their own number of correct answers, iii.) how many correct answers the other group members had individually achieved, iv.) their own profit and v.) the group profit. The group profit increased based on the number of correct calculations by all group members following a step function. Each correct calculation added 5 cents to the group profit with a 50 cent bonus for each tenth correct calculation. This additional bonus is meant to reduce the decline in motivation towards later rounds. The total group profit was equally shared among all group members (own profit = group profit/four). On the next screen the participants had to express how satisfied they were with their own performance (very dissatisfied—very satisfied), how they felt (unpleasant—pleasant) and how high their feeling of arousal was (not aroused—aroused), based on a 9-point SAM (Self-Assessment Manikin) scale [41]. On the next screen, the participants received a detailed list about the performance of all group members and were asked to evaluate them on the same 9-point scale as they evaluated their own performance (very dissatisfied—very satisfied). Then, on the next screen, the participants received feedback on their evaluation from the other group members and again were asked to state their feelings (unpleasant—pleasant) and their feeling of arousal (not aroused—aroused). On the last screen, they were informed about their current total profit.

The duration of the experiment was about 45 minutes. The participants in the “Clinical Sample” filled out the additional questionnaires directly after the experiment. They also had the chance to hand in the questionnaires later, since clinical depression also affects the ability to concentrate and potentially the stress level after such an experiment. Therefore, it would have been irresponsible to keep them in the laboratory for extended periods of time. We allowed both the “Clinically Depressed” and the “Healthy Control” to complete the questionnaires later so that the anonymity of the depressed participants was kept. Nevertheless, all “Healthy Control” participants filled out the questionnaires directly after the experiment. The computer program used for the experiment was the Zurich Toolbox for Readymade Economic Experiments (z-Tree) [42], which is broadly used by experimental economists.

### Procedure single setting

In the “Single” setting, the participants did not interact with other participants and, therefore, received no information or feedback from others. Otherwise, the setting followed the same procedure as mentioned above. The individual measurements of the “Clinically Depressed” group did not take place in the experimental laboratory but in a mobile laboratory consisting of a laptop and a cubicle, which matched in size and appearance with the one in the experimental laboratory.

### Demographic and clinical characteristics

#### Subclinical sample

In total, 339 students participated in our study; 89 had a BDI-II Score above 13 and were categorized as depressed. All demographics and clinical characteristics can be seen in Table 2. The sample sizes in the different settings can be seen in Table 3.

**Table 2:** Demographic and clinical characteristics of subclinical sample.

	Subclinically Depressed	Healthy Control	
**N**	89	250	
**Age (years)**	22.71 (3.95)	23.29 (3.94)	n.s.
**Women (%)**	67.46	50.00	***
**Economist (%)**	27.19	27.20	n.s.
**BDI-II Score**	19.08 (5.01)	6.16 (3.87)	***

Notes: Age: age in years; Women: percentage of women; Economist: percentage of participants having an economics related study field; BDI-II Score: Beck Depression Inventory Score. Two-sided t-test. Standard deviation reported in parenthesis.

*** p<0.01, ** p<0.05, * p<0.1.

**Table 3:** Number of observations in the subclinical sample by treatments.

	Group	Single
**Subclinically Depressed**	31	58
**Healthy**	30	37

#### Clinical sample

In total, we recruited 132 participants; 24 were patients with a treated MDD (“Clinically Depressed”). We only recruited female patients to avoid having to control for gender effects in the group composition of the smaller “Clinical Sample.” The prevalence of MDD is much higher among women [43], and it would have been difficult to balance the composition of workgroups with regard to the sex of the patients included. All demographics and clinical characteristics can be seen in Table 4. The sample sizes in the different settings can be seen in Table 5.

**Table 4:** Demographic and clinical characteristics of clinical sample.

	Clinically Depressed	Healthy Control	
N	24	108	
Age (years)	42.00 (9.67)	35.07 (10.65)	***
High Education (%)	66.66	82.69	*
BDI-II Score	25.05 (10.13)	4.69 (5.19)	***

Notes: Age: age in years; High Education: percentage of people with at least a high school diploma (“Abitur”); BDI-II Score: Beck Depression Inventory Score. Two-sided t-test. Standard deviation reported in parenthesis.

*** p<0.01, ** p<0.05

* p<0.1.

**Table 5:** Number of observations in the clinical sample by treatments.

	Group	Single
**Clinically Depressed**	11	13
**Healthy**	14	19

## Hypotheses

Building on the empirical and theoretical foundations, we derived the following hypotheses. First of all, it can be expected that the overall performance of depressed participants is lower than that of healthy participants [44] caused by cognitive deficits and a lack of energy and motivation. The lower performance of depressed individuals will lead to negative feedback by the remaining group members, starting a vicious cycle. In our experiment, that process should lead to lower and decreasing performance, lower satisfaction with one’s own performance, lower well-being and higher negative arousal for the depressed participants over time.

**Hypothesis 1:** The Group Context negatively influences the performance of depressed participants, as compared to the Individual Context and healthy controls.

**Hypothesis 2:** The Group Context leads to a decrease in performance over time for depressed participants, stronger than the pure motivational effects in the Individual Context and stronger than for healthy controls.

**Hypothesis 3:** The Group Context negatively influences satisfaction in depressed individuals, as compared to the Individual Context and healthy controls.

**Hypothesis 4:** The Group Context negatively influences emotional states in terms of the arousal and well-being of depressed individuals, as compared to the Individual Context and healthy controls.

## Results

### Subclinical sample

#### Performance

As expected, we found that the average performance of the “Subclinically Depressed” (M = 13.01, SD = 3.97) was significantly lower compared to the “Healthy Control” (M = 13.65, SD = 4.04), t(4066) = 4.5, p < 0.001, one-sided t-test. Comparing the performance between “Group” and “Single” treatment, we found a positive effect of the “Group” treatment on the individual performance for “Subclinically Depressed”, t(1066) = -3.9, p < 0.001, one-sided t-test, and “Healthy Control”, t(2998) = -2.0, p = 0.02, one-sided t-test (Table 6).

**Table 6:** Individual performance by treatment.

Correct Calculations	Group (G)	Single (S)	Diff. G–S
**Subclinically**	13.65 (3.85)	12.67 (3.99)	0.98***
**Depressed (D)**			
**Healthy Control (H)**	13.71 (4.13)	13.30 (3.53)	0.41**
**Diff. D–H**	-0.06	-0.64***	

Notes: Average sum of correct calculations in the incentivized periods compared between treatments and types. Two-sided t-test results concern between-group differences. Standard deviation reported in parenthesis.

*** p<0.01

** p<0.05, * p<0.1.

Comparing the performance between the “Subclinically Depressed” and “Healthy Control” we found no difference between them in the “Group” treatment, t(2926) = 0.3, p = 0.77, two-sided t-test, but observed significantly lower performance of the “Subclinically Depressed” compared to the “Healthy Control” in the “Single” treatment, t(1138) = 2.7, p < 0.01, one-sided t-test (Table 6).

We ran a panel regression to see how the performance of the participants developed over time. We found that the performance of the “Subclinically Depressed” was increasing and not decreasing over time (Table 7, (1)–(4)). This increase was even higher in the “Group” treatment (Table 7, (3)-(4)). The performance of the “Healthy Controls” was also increasing over time (Table 7, (1)-(2) & (5)-(6)). Furthermore, we found that the performance of the “Healthy Controls” that were grouped with the “Subclinically Depressed” participants was even higher than for those grouped with other “Healthy Controls,” displaying an additional increase over time (Table 7, (5)-(6)).

**Table 7:** Panel regression on correct calculations in the subclinical sample.

	(1)	(2)	(3)	(4)	(5)	(6)
	All		Subclinically Depressed		Healthy Control	
Dep. Variable	Correct Calculations					
**Group Treatment**	-0.0134	0.154	0.150	0.104	0.178	0.377
	(0.602)	(0.607)	(0.834)	(0.843)	(0.611)	(0.616)
**Period**	0.124***	0.124***	0.0706**	0.0706**	0.124***	0.124***
	(0.0275)	(0.0275)	(0.0287)	(0.0287)	(0.0275)	(0.0275)
**Group Treatment x**	-0.0133	-0.0133	0.0976*	0.0976*	-0.0358	-0.0358
**Period**	(0.0304)	(0.0304)	(0.0530)	(0.0531)	(0.0329)	(0.0329)
**Sub. Depressed**	-0.183	0.270				
	(0.734)	(0.763)				
**Sub. Depressed x**	-1.065	-1.388				
**Group Treatment**	(1.042)	(1.050)				
**Sub. Depressed x**	-0.0533	-0.0533				
**Period**	(0.0396)	(0.0396)				
**Sub. Depressed x**	0.111*	0.111*				
**Group Treatment x Period**	(0.0609)	(0.0609)				
**Healthy Control**	1.229**	1.284***			0.791	0.865*
**w/ Sub. Depressed**	(0.493)	(0.476)			(0.518)	(0.503)
**Healthy Control**					0.0515**	0.0515**
**w/ Sub. Depressed x Period**					(0.0257)	(0.0257)
**Constant**	12.25***	12.44***	12.07***	11.42***	12.25***	12.84***
	(0.501)	(1.304)	(0.539)	(2.631)	(0.501)	(1.427)
**Observations**	4,068	4,068	1,068	1,068	3,000	3,000
**Controls**	No	Yes	No	Yes	No	Yes
**Number of Subjects**	339	339	89	89	250	250

Notes: We report GLS coefficients with standard errors clustered on the individual level in parentheses using a random effects model over 12 periods. The dependent variable is the sum of correct calculations. Controls include dummy variables for male, age and participants having an economics related study field.

*** p<0.01

** p<0.05

* p<0.1.

Looking at the Group Performance (i.e., the sum of the individual performances within a group), we found that the “Subclinically Depressed Group” had a significantly higher performance (M = 56.87, SD = 9.01) compared to the “Healthy Control Group” (M = 52.71, SD = 8.33), t(730) = -6.47, p < 0.001, one-sided t-test. This result was caused by the significantly higher performance of the “Subclinically Depressed” (M = 13.65, SD = 3.85) and “Healthy Controls” (M = 14.41, SD = 4.12) in this setting as compared to the performance of the “Healthy Controls” playing only with other “Healthy Control” (M = 13.18, SD = 4.01), t(1486) = 3.09, p < 0.01 and t(2554) = -7.55, p < 0.001, one-sided t-test (S1 Fig).

#### Satisfaction

The “Subclinically Depressed” indicated, on average, a significantly lower level of satisfaction with their own performance (M = 4.94, SD = 2.03) compared to the “Healthy Controls” (M = 6.21, SD = 2.03), t(4066) = 17.60, p < 0.001, one-sided t-test. In addition, we found a positive effect of the “Group” compared to the “Single” treatment on satisfaction for “Subclinically Depressed”, t(1066) = -4.57, p < 0.001, one-sided t-test, and “Healthy Control”, t(2998) = -2.52, p < 0.01, one-sided t-test (Table 8).

**Table 8:** Individual satisfaction by treatment.

Satisfaction	Group (G)	Single (S)	Diff. G–S
**Subclinically Depressed (D)**	5.33 (2.12)	4.74 (1.95)	0.59***
**Healthy Control (H)**	6.25 (2.06)	5.99 (1.84)	0.26**
**Diff. D–H**	-0.93***	-1.25***	

Notes: Average sum of satisfaction compared between treatment and types using a 9-point Likert Scale from 1 –very unsatisfied to 9 –very satisfied. Two-sided t-test results concern between-group differences. Standard deviation reported in parenthesis.

*** p<0.01

** p<0.05, * p<0.1.

#### Affective states

The “Subclinically Depressed” indicated, on average, a significantly lower level of well-being (M = 4.74, SD = 1.81) compared to “Healthy Controls” (M = 6.21, SD = 1.83), t(4066) = 22.67, p < 0.001, one-sided t-test. The “Subclinically Depressed” in the “Group” treatment indicated a significantly higher level of well-being than those in the “Single” treatment, t(1066) = -4.19, p < 0.001, one-sided t-test. (Table 9). As expected, we found that the “Subclinically Depressed” stated significantly lower levels of well-being compared to “Healthy Controls” in both treatments, Single: t(1138) = 15.1, p < 0.001 and Group: t(2926) = 11.32, p < 0.001, one-sided t-test (Table 9).

**Table 9:** Well-being before feedback by treatment.

Well-Being	Group (G)	Single (S)	Diff. G–S
**Subclinically Depressed (D)**	5.06 (1.84)	4.57 (1.77)	0.48***
**Healthy Control (H)**	6.23 (1.87)	6.12 (1.55)	0.11
**Diff. D–H**	-1.17***	-1.51***	

Notes: Average sum of well-being before feedback compared between treatment and types using a 9-point Likert Scale from 1 –unpleasant to 9 –pleasant. Two-sided t-test results concern between-group differences. Standard deviation reported in parenthesis.

*** p<0.01, ** p<0.05, * p<0.1.

The “Subclinically Depressed” indicated a significantly higher average level of arousal (M = 5.23, SD = 2.11) compared to “Healthy Controls” (M = 4.79, SD = 2.13), t(4066) = -5.84, p < 0.001, one-sided t-test. We also confirmed that the “Subclinically Depressed” stated significantly higher levels of arousal compared to “Healthy Controls” in the “Group” treatment, t(2926) = -6.25, p < 0.001, but not in the “Single” treatment, t(1138) = -0.04, p = 0.48, one-sided t-test. (Table 10). Comparing the arousal between the “Subclinically Depressed” and “Healthy Controls,” we found higher arousal in the “Group” treatment, t(2926) = -6.25, p < 0.001, but no difference in arousal between the “Subclinically Depressed” and “Healthy Controls” in the “Single” treatment, t(1138) = -0.04, p = 0.96, two-sided t-test (Table 10).

**Table 10:** Arousal before feedback by treatment.

Arousal	Group (G)	Single (S)	Diff. G–S
**Subclinically Depressed (D)**	5.47 (1.98)	5.10 (2.16)	0.36***
**Healthy Control (H)**	4.73 (2.10)	5.10 (2.15)	- 0.37***
**Diff. D–H**	0.65***	0.00	

Notes: Average sum of well-being before feedback compared between treatment and types using a 9-point Likert Scale from 1 –unexcited to 9 –excited. Two-sided t-test results concern between-group differences. Standard deviation reported in parenthesis.

*** p<0.01, ** p<0.05, * p<0.1.

### Clinical sample

#### Performance

Again, as expected, we found that on average the performance of the “Clinically Depressed” (M = 8.74, SD = 3.35) was significantly lower compared to the performance of “Healthy Controls” (M = 11.18, SD = 3.75), t(1582) = 10.17, p < 0.001, one-sided t-test. Comparing the performance between “Group” and “Single” treatments, we found no effect on the individual performance of the”Clinically Depressed” and the “Healthy Controls”, t(286) = 0.41, p = 0.68 and t(1294) = -1.13, p = 0.26 two-sided t-test. (Table 11). Comparing the performance between the “Clinically Depressed” and “Healthy Controls” we found a lower performance of the “Clinically Depressed” compared to “Healthy Controls” for both treatments, Single: t(382) = 6.63, p < 0.001 and Group: t(1198) = 7.25, p < 0.001, one-sided t-test. (Table 11).

**Table 11:** Individual performance by treatment.

Correct Calculations	Group (G)	Single (S)	Diff. G–S
**Clinically Depressed (P)**	8.65 (3.45)	8.81 (3.26)	-0.16
**Healthy Control (HC)**	11.23(3.91)	10.93 (2.92)	0.31
**Diff. P–HC**	-2.58***	-2.11***	

Notes: Average sum of correct calculations in the payment periods compared between treatments and types. Two-sided t-test results concern between-group differences. Standard deviation reported in parenthesis.

*** p<0.01, ** p<0.05, * p<0.1.

We ran a panel regression to see how the performance of the participants developed over time. We found that the performance of the “Clinically Depressed” was lower compared to the “Healthy Controls” but increased over time in contrast to our expectations, but in line with the findings in the “Subclinical Sample” (Table 12).

**Table 12:** Panel regression on correct calculations in the clinical sample.

	(1)	(2)	(3)	(4)	(5)	(6)
	All		Clinically Depressed		Healthy Control	
Dep. Variable	Correct Calculations					
**Group Treatment**	0.405	0.166	0.313	1.195	0.431	0.161
	(0.731)	(0.646)	(1.336)	(1.321)	(0.743)	(0.661)
**Period**	0.173***	0.173***	0.183***	0.196***	0.173***	0.173***
	(0.0431)	(0.0432)	(0.0275)	(0.0283)	(0.0431)	(0.0432)
**Group Treatment x**	-0.0330	-0.0343	-0.0732	-0.0849	-0.0369	-0.0369
**Period**	(0.0467)	(0.0468)	(0.0520)	(0.0565)	(0.0497)	(0.0497)
**Clin. Depressed**	-2.179**	-1.866***				
	(0.986)	(0.715)				
**Clin. Depressed x**	-0.413	0.868				
**Group Treatment**	(1.512)	(1.437)				
**Clin. Depressed x Period**	0.0104	0.0236				
	(0.0509)	(0.0512)				
**Clin. Depressed x**	-0.0402	-0.0506				
**Group Treatment x Period**	(0.0691)	(0.0722)				
**Healthy Control w/ Clin.**	0.321	0.123			0.251	0.114
**Depressed**	(0.719)	(0.752)			(0.788)	(0.821)
**Healthy Control w/ Clin.**					0.0107	0.00785
**Depressed x Period**					(0.0343)	(0.0343)
**Constant**	9.802***	7.825***	7.624***	1.112	9.802***	8.937***
	(0.518)	(2.094)	(0.856)	(2.646)	(0.518)	(2.389)
**Observations**	1,584	1,500	288	252	1,296	1,248
**Controls**	No	Yes	No	Yes	No	Yes
**Number of Subjects**	132	125	24	21	108	104

Notes: We report GLS coefficients with standard errors clustered on the individual level in parentheses using a random effects model over 12 periods. The dependent variable is the sum of correct calculations. Controls include dummy variables for education and age.

*** p<0.01

** p<0.05, * p<0.1.

Looking at the Group Performance, we found that, on average, the “Clinically Depressed Group” (M = 42.96, SD = 8.86) had a significantly lower performance compared to the “Healthy Group” (M = 44.46, SD = 9.14), t(298) = 1.43, p = 0.08, one-sided t-test.

#### Satisfaction

As expected, the “Clinically Depressed” indicated, on average, a significantly lower level of satisfaction (M = 5.32, SD = 2.53) compared to the “Healthy Controls” (M = 6.25, SD = 2.06), t(286) = 0.41, p = 0.68, one-sided t-test). In contrast to our hypothesis, but in line with the findings in the subclinical sample, we found a positive effect of the “Group” compared to the “Single” treatment on satisfaction for “Clinically Depressed”, t(286) = -2.23, p = 0.01, and the “Healthy Controls”, t(1294) = -3.97, p < 0.001, one-sided t-test (Table 13).

**Table 13:** Individual satisfaction by treatment.

Satisfaction	Group (G)	Single (S)	Diff. G–S
**Clinically**	5.68 (2.45)	5.02 (2.55)	0.66**
**Depressed (P)**			
**Healthy Control (HC)**	6.36 (2.07)	5.76 (1.97)	0.60***
**Diff. P–HC**	-0.68***	-0.74***	

Notes: Average sum of satisfaction compared between treatment and types using a 9-point Likert Scale from 1 –very unsatisfied to 9 –very satisfied. Two-sided t-test results concern between-group differences. Standard deviation reported in parenthesis.

*** p<0.01

** p<0.05, * p<0.1.

#### Affective states

“Clinically Depressed” indicated a significantly lower level of well-being (M = 5.38, SD = 2.47) compared to “Healthy Controls” (M = 6.19, SD = 1.93), t(1582) = 6.14, p < 0.001, one-sided t-test. We found no differences in well-being for the “Clinically Depressed” between the treatments, t(286) = 0.55, p = 0.58, two-sided t-test, and a positive effect of the group treatment on the “Healthy Controls”, t(1294) = -1.70, p = 0.04, one-sided t-test. (Table 14). We found that the “Clinically Depressed” stated significantly lower levels of well-being compared to the “Healthy Control” in both treatments, Single: t(382) = 2.41, p < 0.01 and Group: t(1198) = 5.13, p < 0.001, one-sided t-test (Table 14).

**Table 14:** Well-being before feedback.

Well-Being	Group (G)	Single (S)	Diff. G–S
**Clinically**	5.29 (2.31)	5.45 (2.59)	-0.16
**Depressed (P)**			
**Healthy Control (HC)**	6.23 (1.95)	5.99 (1.82)	0.24*
**Diff. P–HC**	-0.94***	-0.54**	

Notes: Average sum of well-being before feedback compared between treatment and types using a 9-point Likert Scale from 1 –unpleasant to 9 –pleasant. Two-sided t-test results concern between-group differences. Standard deviation reported in parenthesis.

*** p<0.01

** p<0.05

* p<0.1.

The “Clinically Depressed” indicated on average a significantly higher level of arousal (M = 5.89, SD = 2.05) compared to the “Healthy Controls” (M = 4.91, SD = 2.21), t(1582) = -6.78, p < 0.001, one-sided t-test. As hypothesized, the “Clinically Depressed” in the “Group” treatment indicated a significantly higher level of arousal than those in the “Single” treatment), t(286) = -4.40, p < 0.001, one-sided t-test (Table 15). We found that the “Clinically Depressed” stated significantly higher levels of arousal compared to the “Healthy Control” in both treatments, Single: t(382) = -2.39, p < 0.01 and Group: t(1198) = -7.43, p < 0.001, one-sided t-test (Table 15).

**Table 15:** Arousal before feedback by treatment.

Arousal	Group (G)	Single (S)	Diff. G–S
**Clinically Depressed (P)**	6.44 (1.97)	5.40 (2.01)	1.04***
**Healthy Control (HC)**	4.92 (2.25)	4.90 (2.02)	0.02
**Diff. P–H**	1.52***	0.50**	

Notes: Average sum of well-being before feedback compared between treatment and types using a 9-point Likert Scale from 1 –unexcited to 9 –excited. Two-sided t-test results concern between-group differences. Standard deviation reported in parenthesis.

*** p<0.01

** p<0.05

* p<0.1.

## Conclusion and discussion

The aim of this project was to use a controlled laboratory setting to test how the *interpersonal context* of the workplace—working in a group or individually—impacts the mood and productivity of depressed and non-depressed workers and the teams they work in. In two studies, healthy and depressed individuals solved real-effort tasks, either in a stylized group context or in an individual working context. Groups were comprised of either only healthy subjects or healthy subjects and one depressed participant. The two studies differed in the sample used: i.) a random student population divided into a subclinically depressed and healthy subgroup and ii.) a clinical sample of depressed individuals at the end of an inpatient or day-clinic treatment and matched to healthy controls. These two samples were chosen to understand both the influence of the work context of those on the brink of depression and on those returning to the workplace after a depressive episode. Based on the assumption that the cognitive performance of depressed participants is lower than that of healthy individuals, we hypothesized that the context would have specific effects on performance and mood in the different settings over the time of the experiment. We expected the group context to have negative effects on the performance, satisfaction and mood of the depressed participants and potentially their group members.

The results show the expected lower performance of depressed participants compared to healthy participants within each of the samples. However, in contrast to our expectations, the performance of subclinically depressed individuals working in groups with healthy controls is even higher than the performance of healthy controls in homogenously healthy control groups. In addition, a positive performance effect of working in a group is found in the student sample for both healthy and subclinically depressed individuals. We observe no decrease in performance of groups with subclinically depressed participants over time.

Thus, we neither confirm our *first hypothesis* that being in a group context negatively affects performance of depressed participants in general, nor our *second hypothesis*, which predicted a decrease in performance over time.

The difference in the performance effects of depression in the two samples can most likely be attributed to the difference in the severity of depression. Students have a significantly lower depression severity (i.e., average BDI scores of 19.08 (5.01) among students vs 25.05 (10.13) in the clinically depressed group, p<0.001 one-sided t-test), which might account for the preserved ability to increase their performance in the group context. Such a link between depression severity and cognitive performance is supported by various studies [13, 14]. Clinically depressed individuals show reduced cognitive performance at baseline but not an impaired training effect or exhaustion during the task. In contrast, the performance increase of the subclinically depressed students is still even high enough to let their whole group surpass the performance of groups without a depressed participant.

Another factor explaining the difference in performance between the student sample and the clinically depressed sample is the higher overall performance level and the lower average age in the student sample. Both factors could provide higher cognitive baseline resources as a resilience factor to compensate for cognitive impairments associated with depression. Thus, we confirm through our experiments that depression impairs cognitive resources, which is reflected in performance reduction in an individual work context. The group context can compensate for this impairment if i.) depression severity is not too high and ii.) baseline performance/cognitive resources provide resilience to compensate for the task.

Our *third hypothesis* claimed that the group context negatively influences satisfaction in depressed individuals; this, however, is not confirmed by our experiments. On the contrary, the overall work satisfaction is generally higher in groups for both samples, the student sample and the clinical sample.

Concerning the *fourth hypothesis*, we find mixed results. While the group context had a positive effect on the self-reported well-being of the subclinically depressed participants and no effect on the well-being of the clinically depressed participants, we could confirm a negative effect of being in a group on arousal in both samples. The functional model of depression by Holtzheimer and Mayberg [45] has characterized the development of major depression as a failure of homeostatic interaction with the social environment. It is suggested that “depression is better defined as the tendency to enter into, and inability to disengage from, a negative mood state rather than the mood state per se” [45]. In this concept, depression is defined by a deficit of homeostatic self-regulation when facing stressful life events with increased proneness to enter and stay in negative mood states. It seems that the group context could at least partially alleviate this.

The positive effects of a group context which we find in the student sample, underlines the idea that these may be restricted to less depressive and younger individuals with greater resources to compensate for the cognitive effects of depression. The assumption that not only depression severity but also age is associated with reduced motivational and affective flexibility is supported by recent empirical data [e.g., 46, 47].

While, to the best of our knowledge, this study is the first to systematically compare the effects of different kinds of work organization on (sub)clinically depressed workers and their teams, it also has some limitations. First, the sample of individuals suffering from a diagnosed major depressive disorder is relatively small and consists of only women, whereas the sample of subclinically depressed participants is comprised of male and female students, i.e., the two samples differ in sex, age and education level. For practical reasons, as the population of clinically depressed participants available for research is small and diagnosed major depression is more frequent among women, it was not feasible to match the clinical sample with the student sample in size and demographics. Further studies, including patient recruitment in multiple centers, are necessary to figure out how generalizable the results from the small all-female patient samples are to the general population of the clinically depressed.

The issue of generalizability also affects our stylized work setting. Of course, working in a real team setting differs in many respects from a laboratory real-effort task in a group that is only connected by the interdependency of the earnings and the possibility to send each other standardized feedback. While this covers important aspects of group-based work settings and allows for controlled variation of them, further research should expand this towards more realistic (field) settings to understand the relative importance of the factors discussed here, and the role other aspects of a true team setting play.

Another question that our experiment cannot solve is whether the positive effects of being in a group are sustainable for both sides. Our results reflect only a relatively short interaction, in which the healthy controls partially overcompensate for the reduced performance of the depressed team members; this might be a strategy that healthy team members cannot and do not want to sustain over larger periods. Thus, it remains to be studied whether these positive effects of group work on both the well-being and performance of depressed group members are sustainable over the long run. The outcomes clearly depend on the severity of depression, but—in our setting—all depressed group members profit from or at least do not worsen in well-being and performance in a group setting.

Notably, enabling work in the group context is not only valuable advice in an attempt to preserve the resilience of a companies’ workforce but also from the individual employees’ perspective. Working in a group also preserves individual well-being. Thus, both from the perspective of the employer and that of the employee, it is important to make sure that people at risk for or recovering from depression are integrated into work teams to preserve their ability to work and to avoid relapse.

While some questions need to be addressed by future research, we can already recommend that mental healthcare and management strategies integrate a deliberate choice of work context allowing for integration into teams of healthy workers to improve rehabilitation.

## Supporting information

S1 Fig Individual performance by treatment and type, subclinical sample. (TIF)

S2 Fig Individual performance by treatment and type, clinical sample. (TIF)

S1 Table Panel regression on satisfaction in the subclinical sample. (DOCX)

S2 Table Panel regression on well-being in the subclinical sample. (DOCX)

S3 Table Panel regression on arousal in the subclinical sample. (DOCX)

S4 Table Panel regression on satisfaction in the clinical sample. (DOCX)

S5 Table Panel regression on well-being in the clinical sample. (DOCX)

S6 Table Panel regression on arousal in the clinical sample. (DOCX)
