# Supplementary figures and images for: Let the team fix it?—Performance and mood of depressed workers and coworkers in different work contexts
Source: PLoS One. 2021 Oct 14;16(10):e0256553. doi: 10.1371/journal.pone.0256553 (PMC8516233; doi:10.1371/journal.pone.0256553)

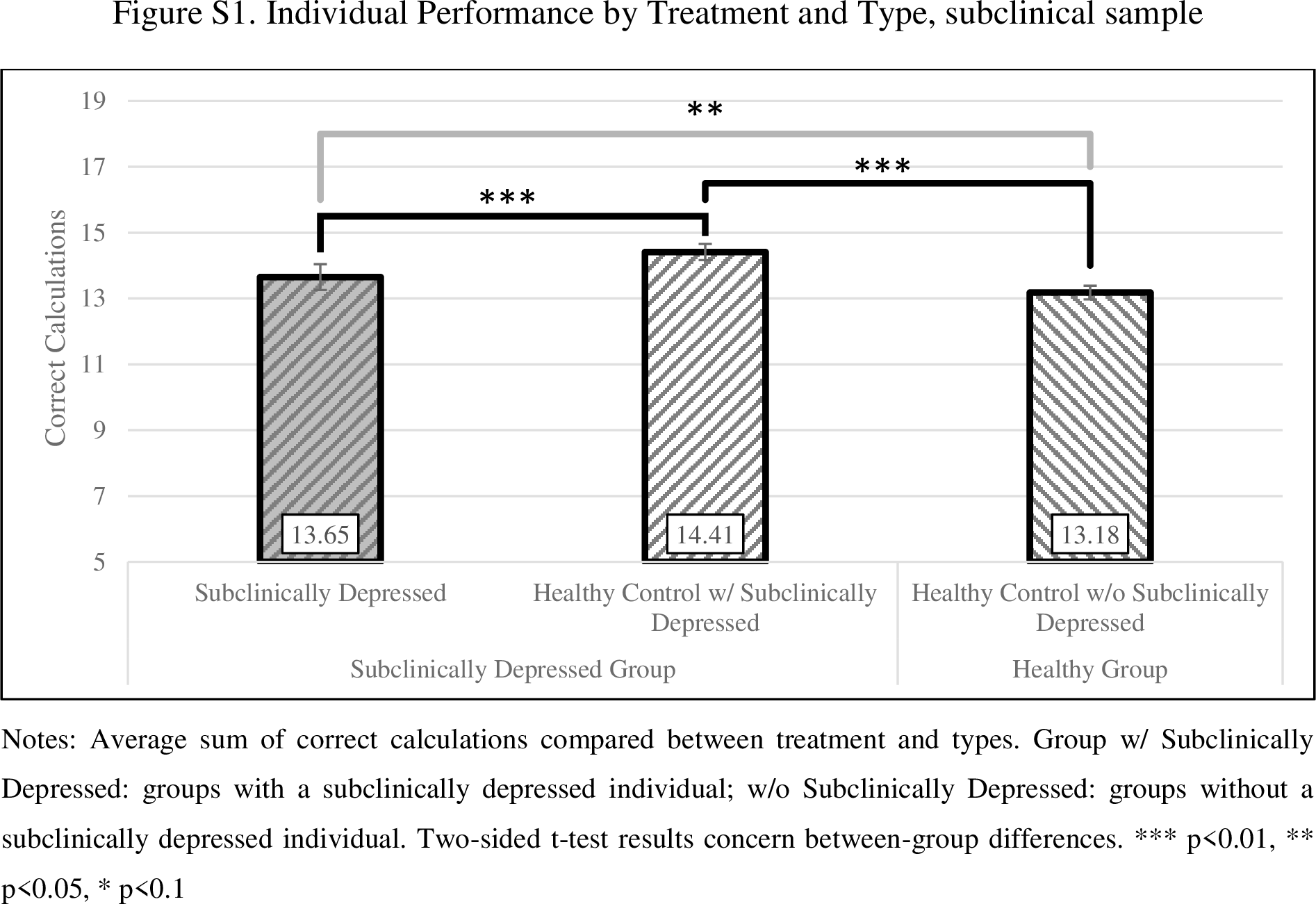

Supplement: S1 Fig — (TIF) [file pone.0256553.s001.tif]

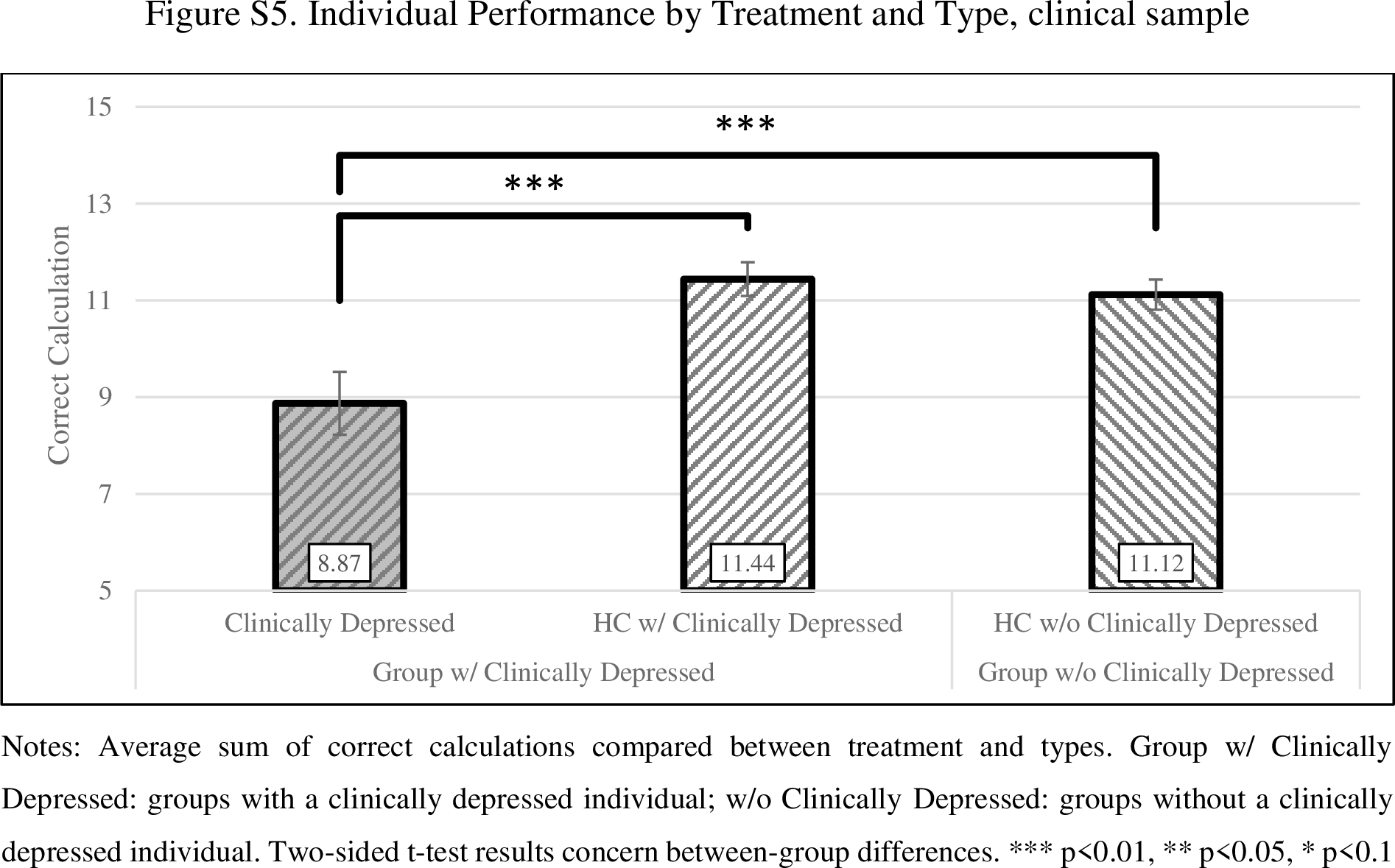

Supplement: S2 Fig — (TIF) [file pone.0256553.s002.tif]
