# Supplementary material for: Let the team fix it?—Performance and mood of depressed workers and coworkers in different work contexts
Source: PLoS One. 2021 Oct 14;16(10):e0256553. doi: 10.1371/journal.pone.0256553 (PMC8516233; doi:10.1371/journal.pone.0256553)
Supplement: S1 Table — (DOCX) [file pone.0256553.s003.docx]

S1 Table. Panel Regression on Satisfaction in the Subclinical Sample

|  | (1) | (2) | (3) | (4) | (5) | (6) |
| --- | --- | --- | --- | --- | --- | --- |
|  | All | | Subclinically Depressed | | Healthy Control | |
| Dep. Variable | Satisfaction | | | | | |
| Group Treatment | 0.354 | 0.361 | 0.439 | 0.420 | 0.357 | 0.356 |
|  | (0.323) | (0.324) | (0.454) | (0.449) | (0.329) | (0.329) |
| Period | 0.0253 | 0.0253 | -0.0271 | -0.0271 | 0.0253 | 0.0253 |
|  | (0.0264) | (0.0264) | (0.0195) | (0.0196) | (0.0264) | (0.0264) |
| Group Treatment x | -0.0142 | -0.0142 | 0.0178 | 0.0178 | -0.0144 | -0.0144 |
| Period | (0.0278) | (0.0278) | (0.0376) | (0.0377) | (0.0291) | (0.0291) |
| Sub. Depressed | -0.809** | -0.771** |  |  |  |  |
|  | (0.378) | (0.383) |  |  |  |  |
| Sub. Depressed x | 0.0197 | 0.0152 |  |  |  |  |
| Group Treatment | (0.555) | (0.556) |  |  |  |  |
| Sub. Depressed x | -0.0524 | -0.0524 |  |  |  |  |
| Period | (0.0328) | (0.0328) |  |  |  |  |
| Sub. Depressed x | 0.0320 | 0.0320 |  |  |  |  |
| Group Treatment x Period | (0.0466) | (0.0467) |  |  |  |  |
| Healthy Control | 0.0653 | 0.0598 |  |  | 0.0602 | 0.0452 |
| w/ Sub. Depressed | (0.211) | (0.211) |  |  | (0.243) | (0.242) |
| Healthy Control |  |  |  |  | 0.000607 | 0.000607 |
| w/Sub. Depressed x Period |  |  |  |  | (0.0175) | (0.0175) |
| Constant | 5.776*** | 5.512*** | 4.967*** | 5.308*** | 5.776*** | 5.319*** |
|  | (0.279) | (0.501) | (0.256) | (0.900) | (0.279) | (0.600) |
| Observations | 4,068 | 4,068 | 1,068 | 1,068 | 3,000 | 3,000 |
| Controls | No | Yes | No | Yes | No | Yes |
| Number of Subjects | 339 | 339 | 89 | 89 | 250 | 250 |

Notes: We report GLS coefficients with standard errors clustered on the individual level in parentheses using a random effects model over 12 periods. The dependent variable is the level of satisfaction. Controls include dummy variables for education and age. *** p<0.01, ** p<0.05, * p<0.
