# Supplementary material for: Let the team fix it?—Performance and mood of depressed workers and coworkers in different work contexts
Source: PLoS One. 2021 Oct 14;16(10):e0256553. doi: 10.1371/journal.pone.0256553 (PMC8516233; doi:10.1371/journal.pone.0256553)
Supplement: S2 Table — (DOCX) [file pone.0256553.s004.docx]

S2 Table. Panel Regression on Well-Being in the Subclinical Sample

|  | (1) | (2) | (3) | (4) | (5) | (6) |
| --- | --- | --- | --- | --- | --- | --- |
|  | All | | Subclinically Depressed | | Healthy Control | |
| Dep. Variable | Well-Being | | | | | |
| Group Treatment | 0.255 | 0.264 | 0.285 | 0.260 | 0.263 | 0.263 |
|  | (0.306) | (0.307) | (0.388) | (0.371) | (0.310) | (0.311) |
| Period | 0.0265 | 0.0265 | -0.0401** | -0.0401** | 0.0265 | 0.0265 |
|  | (0.0250) | (0.0250) | (0.0183) | (0.0184) | (0.0250) | (0.0250) |
| Group Treatment x | -0.0194 | -0.0194 | 0.0233 | 0.0233 | -0.0205 | -0.0205 |
| Period | (0.0263) | (0.0263) | (0.0340) | (0.0341) | (0.0273) | (0.0273) |
| Sub. Depressed | -0.983*** | -0.933*** |  |  |  |  |
|  | (0.344) | (0.349) |  |  |  |  |
| Sub. Depressed x | -0.0135 | -0.0223 |  |  |  |  |
| Group Treatment | (0.493) | (0.490) |  |  |  |  |
| Sub. Depressed x | -0.0665** | -0.0665** |  |  |  |  |
| Period | (0.0309) | (0.0309) |  |  |  |  |
| Group Treatment x | 0.0427 | 0.0427 |  |  |  |  |
| Sub. Depressed x Period | (0.0428) | (0.0429) |  |  |  |  |
| Healthy Control | 0.0443 | 0.0385 |  |  | 0.0240 | 0.00730 |
| w/ Sub. Depressed | (0.212) | (0.212) |  |  | (0.235) | (0.236) |
| Healthy Control |  |  |  |  | 0.00238 | 0.00238 |
| w/ Sub. Depressed x Period |  |  |  |  | (0.0164) | (0.0164) |
| Constant | 5.897*** | 5.585*** | 4.914*** | 5.278*** | 5.897*** | 5.379*** |
|  | (0.261) | (0.506) | (0.226) | (0.863) | (0.261) | (0.635) |
| Observations | 4,068 | 4,068 | 1,068 | 1,068 | 3,000 | 3,000 |
| Controls | No | Yes | No | Yes | No | Yes |
| Number of Subjects | 339 | 339 | 89 | 89 | 250 | 250 |

Notes: We report GLS coefficients with standard errors clustered on the individual level in parentheses using a random effects model over 12 periods. The dependent variable is the level of well-being. Controls include dummy variables for education and age. *** p<0.01, ** p<0.05, * p<0.1
