# Supplementary material for: Let the team fix it?—Performance and mood of depressed workers and coworkers in different work contexts
Source: PLoS One. 2021 Oct 14;16(10):e0256553. doi: 10.1371/journal.pone.0256553 (PMC8516233; doi:10.1371/journal.pone.0256553)
Supplement: S3 Table — (DOCX) [file pone.0256553.s005.docx]

S3 Table. Panel Regression on Arousal in the Subclinical Sample

|  | (1) | (2) | (3) | (4) | (5) | (6) |
| --- | --- | --- | --- | --- | --- | --- |
|  | All | | Subclinically Depressed | | Healthy Control | |
| Dep. Variable | Arousal | | | | | |
| Group Treatment | -0.119 | -0.0939 | 0.599 | 0.571 | -0.142 | -0.123 |
|  | (0.405) | (0.408) | (0.505) | (0.506) | (0.410) | (0.415) |
| Period | 0.0161 | 0.0161 | 0.0260 | 0.0260 | 0.0161 | 0.0161 |
|  | (0.0195) | (0.0195) | (0.0216) | (0.0216) | (0.0195) | (0.0195) |
| Group Treatment x | -0.0455** | -0.0455** | -0.0281 | -0.0281 | -0.0428* | -0.0428* |
| Period | (0.0216) | (0.0216) | (0.0428) | (0.0429) | (0.0231) | (0.0231) |
| Sub. Depressed | -0.0785 | -0.0316 |  |  |  |  |
|  | (0.455) | (0.463) |  |  |  |  |
| Sub. Depressed x | 0.395 | 0.339 |  |  |  |  |
| Group Treatment | (0.644) | (0.652) |  |  |  |  |
| Sub. Depressed x | 0.00992 | 0.00992 |  |  |  |  |
| Period | (0.0290) | (0.0290) |  |  |  |  |
| Sub. Depressed x | 0.0174 | 0.0174 |  |  |  |  |
| Group Treatment x Period | (0.0478) | (0.0478) |  |  |  |  |
| Healthy Control | 0.323 | 0.339 |  |  | 0.375 | 0.371 |
| w/ Sub. Depressed | (0.261) | (0.264) |  |  | (0.287) | (0.288) |
| Healthy Control |  |  |  |  | -0.00618 | -0.00618 |
| w/ Sub. Depressed x Period |  |  |  |  | (0.0192) | (0.0192) |
| Constant | 4.963*** | 5.262*** | 4.884*** | 6.726*** | 4.963*** | 4.703*** |
|  | (0.354) | (0.729) | (0.287) | (1.326) | (0.355) | (0.684) |
| Observations | 4,068 | 4,068 | 1,068 | 1,068 | 3,000 | 3,000 |
| Controls | No | Yes | No | Yes | No | Yes |
| Number of Subjects | 339 | 339 | 89 | 89 | 250 | 250 |

Notes: We report GLS coefficients with standard errors clustered on the individual level in parentheses using a random effects model over 12 periods. The dependent variable is the level of arousal. Controls include dummy variables for education and age. *** p<0.01, ** p<0.05, * p<0.1
