# Supplementary material for: Let the team fix it?—Performance and mood of depressed workers and coworkers in different work contexts
Source: PLoS One. 2021 Oct 14;16(10):e0256553. doi: 10.1371/journal.pone.0256553 (PMC8516233; doi:10.1371/journal.pone.0256553)
Supplement: S4 Table — (DOCX) [file pone.0256553.s006.docx]

S4 Table. Panel Regression on Satisfaction in the Clinical Sample

|  | (1) | (2) | (3) | (4) | (5) | (6) |
| --- | --- | --- | --- | --- | --- | --- |
|  | All | | Clinically Depressed | | Healthy Control | |
| Dep. Variable | Satisfaction | | | | | |
| Group Treatment | 0.288 | 0.212 | 0.693 | 1.225 | 0.247 | 0.209 |
|  | (0.524) | (0.529) | (0.901) | (0.971) | (0.529) | (0.531) |
| Period | 0.0210 | 0.0210 | 0.00726 | 0.0124 | 0.0210 | 0.0210 |
|  | (0.0383) | (0.0383) | (0.0260) | (0.0306) | (0.0383) | (0.0383) |
| Group Treatment x | 0.0216 | 0.0255 | -0.00472 | 0.00579 | 0.0280 | 0.0280 |
| Period | (0.0415) | (0.0417) | (0.0530) | (0.0572) | (0.0439) | (0.0439) |
| Clin. Depressed | -0.655 | -0.718 |  |  |  |  |
|  | (0.782) | (0.719) |  |  |  |  |
| Clin. Depressed x | -0.0420 | 0.278 |  |  |  |  |
| Group Treatment | (1.027) | (1.062) |  |  |  |  |
| Clin. Depressed x | -0.0137 | -0.00858 |  |  |  |  |
| Period | (0.0460) | (0.0485) |  |  |  |  |
| Clin. Depressed x | -0.0263 | -0.0198 |  |  |  |  |
| Group Treatment x Period | (0.0665) | (0.0696) |  |  |  |  |
| Healthy Control | 0.447 | 0.465 |  |  | 0.560* | 0.563 |
| w/ Clin. Depressed | (0.288) | (0.293) |  |  | (0.339) | (0.368) |
| Healthy Control |  |  |  |  | -0.0173 | -0.00730 |
| w/ Clin. Depressed x Period |  |  |  |  | (0.0314) | (0.0332) |
| Constant | 5.627*** | 5.168*** | 4.972*** | 3.066 | 5.627*** | 5.723*** |
|  | (0.475) | (1.239) | (0.634) | (4.056) | (0.475) | (1.199) |
| Observations | 1,584 | 1,500 | 288 | 252 | 1,296 | 1,248 |
| Controls | No | Yes | No | Yes | No | Yes |
| Number of Subjects | 132 | 125 | 24 | 21 | 108 | 104 |

Notes: We report GLS coefficients with standard errors clustered on the individual level in parentheses using a random effects model over 12 periods. The dependent variable is the level of satisfaction. Controls include dummy variables for education and age. *** p<0.01, ** p<0.05, * p<0.1
