# Supplementary material for: Let the team fix it?—Performance and mood of depressed workers and coworkers in different work contexts
Source: PLoS One. 2021 Oct 14;16(10):e0256553. doi: 10.1371/journal.pone.0256553 (PMC8516233; doi:10.1371/journal.pone.0256553)
Supplement: S5 Table — (DOCX) [file pone.0256553.s007.docx]

S5 Table. Panel Regression on Well-Being in the Clinical Sample

|  | (1) | (2) | (3) | (4) | (5) | (6) |
| --- | --- | --- | --- | --- | --- | --- |
|  | All | | Clinically Depressed | | Healthy Control | |
| Dep. Variable | Well-Being | | | | | |
| Group Treatment | 0.0942 | 0.0511 | -0.131 | 0.623 | 0.0699 | 0.0703 |
|  | (0.470) | (0.480) | (0.924) | (0.999) | (0.470) | (0.473) |
| Period | 0.0118 | 0.0118 | -0.0355 | -0.0420 | 0.0118 | 0.0118 |
|  | (0.0229) | (0.0229) | (0.0231) | (0.0270) | (0.0229) | (0.0230) |
| Group Treatment x | 0.00952 | 0.0132 | -0.00455 | 0.000350 | 0.0133 | 0.0133 |
| Period | (0.0264) | (0.0266) | (0.0455) | (0.0511) | (0.0286) | (0.0286) |
| Clin. Depressed | -0.235 | -0.703 |  |  |  |  |
|  | (0.782) | (0.768) |  |  |  |  |
| Clin. Depressed x | -0.449 | 0.0875 |  |  |  |  |
| Group Treatment | (1.022) | (1.082) |  |  |  |  |
| Clin. Depressed x | -0.0473 | -0.0537 |  |  |  |  |
| Period | (0.0322) | (0.0349) |  |  |  |  |
| Clin. Depressed x | -0.0141 | -0.0128 |  |  |  |  |
| Group Treatment x Period | (0.0518) | (0.0564) |  |  |  |  |
| Healthy Control | 0.224 | 0.208 |  |  | 0.290 | 0.252 |
| w/ Clin. Depressed | (0.325) | (0.331) |  |  | (0.334) | (0.351) |
| Healthy Control |  |  |  |  | -0.0101 | -0.000321 |
| w/ Clin. Depressed x Period |  |  |  |  | (0.0265) | (0.0275) |
| Constant | 5.915*** | 5.582*** | 5.679*** | 4.711 | 5.915*** | 5.873*** |
|  | (0.411) | (1.375) | (0.679) | (4.562) | (0.411) | (1.362) |
| Observations | 1,584 | 1,500 | 288 | 252 | 1,296 | 1,248 |
| Controls | No | Yes | No | Yes | No | Yes |
| Number of Subjects | 132 | 125 | 24 | 21 | 108 | 104 |

Notes: We report GLS coefficients with standard errors clustered on the individual level in parentheses using a random effects model over 12 periods. The dependent variable is the level of well-being. Controls include dummy variables for education and age. *** p<0.01, ** p<0.05, * p<0.1
