# Supplementary material for: Let the team fix it?—Performance and mood of depressed workers and coworkers in different work contexts
Source: PLoS One. 2021 Oct 14;16(10):e0256553. doi: 10.1371/journal.pone.0256553 (PMC8516233; doi:10.1371/journal.pone.0256553)
Supplement: S6 Table — (DOCX) [file pone.0256553.s008.docx]

S6 Table. Panel Regression on Arousal in the Clinical Sample

|  | (1) | (2) | (3) | (4) | (5) | (6) |
| --- | --- | --- | --- | --- | --- | --- |
|  | All | | Clinically Depressed | | Healhty Cotnrol | |
| Dep. Variable | Arousal | | | | | |
| Group Treatment | 0.228 | 0.114 | 0.575 | -0.185 | 0.169 | 0.0554 |
|  | (0.571) | (0.576) | (0.896) | (0.906) | (0.571) | (0.577) |
| Period | -0.00626 | -0.00626 | 0.0137 | 0.0162 | -0.00626 | -0.00626 |
|  | (0.0408) | (0.0409) | (0.0485) | (0.0577) | (0.0409) | (0.0409) |
| Group Treatment x | -0.00675 | -0.00596 | 0.0708 | 0.0824 | 0.00232 | 0.00232 |
| Period | (0.0443) | (0.0446) | (0.0606) | (0.0688) | (0.0470) | (0.0471) |
| Clin. Depressed | 0.371 | 0.906 |  |  |  |  |
|  | (0.791) | (0.794) |  |  |  |  |
| Clin. Depressed x | 0.809 | 0.243 |  |  |  |  |
| Group Treatment | (1.065) | (1.104) |  |  |  |  |
| Clin. Depressed x | 0.0200 | 0.0225 |  |  |  |  |
| Period | (0.0626) | (0.0695) |  |  |  |  |
| Clin. Depressed x | 0.0776 | 0.0884 |  |  |  |  |
| Group Treatment x Period | (0.0741) | (0.0804) |  |  |  |  |
| Healthy Control | -0.462 | -0.364 |  |  | -0.303 | -0.245 |
| w/ Clin. Depressed | (0.428) | (0.445) |  |  | (0.440) | (0.451) |
| Healthy Control |  |  |  |  | -0.0245 | -0.0243 |
| w/ Clin. Depressed x Period |  |  |  |  | (0.0334) | (0.0349) |
| Constant | 4.944*** | 6.212*** | 5.315*** | 8.762** | 4.944*** | 5.683*** |
|  | (0.501) | (1.571) | (0.626) | (3.866) | (0.501) | (1.674) |
| Observations | 1,584 | 1,500 | 288 | 252 | 1,296 | 1,248 |
| Controls | No | Yes | No | Yes | No | Yes |
| Number of Subjects | 132 | 125 | 24 | 21 | 108 | 104 |

Notes: We report GLS coefficients with standard errors clustered on the individual level in parentheses using a random effects model over 12 periods. The dependent variable is the level of arousal. Controls include dummy variables for education and age. *** p<0.01, ** p<0.05, * p<0.1
